# In Vitro Evaluation and Network Pharmacology Analysis of the Antimicrobial Activity of *Pistacia lentiscus*

**DOI:** 10.1155/ijod/6981413

**Published:** 2026-01-20

**Authors:** Aparna Ganeshkumar, Ram Sabarish, Nadha Shakir, Divya Shree Sankar, Adelyn Jerusha Franklin, Nagalakshmi Gandhi, Kennedy Kumar

**Affiliations:** ^1^ Department of Periodontics, Sri Ramachandra Dental College and Hospital, Sri Ramachandra Institute of Higher Education and Research, Chennai, India, sriramachandra.edu.in; ^2^ Department of Microbiology, Sri Ramachandra Medical College and Hospital, Sri Ramachandra Institute of Higher Education and Research, Chennai, India, sriramachandra.edu.in

**Keywords:** anti-infective agents, antimicrobial resistance, drug discovery, network pharmacology, phytotherapy

## Abstract

**Background:**

Chios mastic gum, a natural resin derived from the mastic tree, has a history of traditional use for its beneficial effects on the gastrointestinal system, its anti‐inflammatory properties, and its antimicrobial activity.

**Objective:**

This study aimed to integrate network pharmacology and standardized *in vitro* analyses to elucidate the antimicrobial mechanisms and therapeutic potential of *P. lentiscus* phytochemicals.

**Material and Methods:**

This study employed a systematic methodology integrating data mining, network construction, and network analysis to explore the intricate relationships between the constituents of mastic gum and their diverse biological targets. The primary active compounds of *P. lentiscus* were identified through multiple databases, including the Indian Medicinal Plants, Phytochemistry, and Therapeutics database. ADME/T profiling, Lipinski’s rule of five, and target prediction were performed to assess drug likeness and pharmacokinetics. Protein–protein interaction networks and gene ontology (GO) enrichment were analyzed using STRING and Cytoscape to identify biological pathways associated with antimicrobial activity. Moreover, the antibacterial efficacy of these compounds was evaluated following the Polish‐European standard 1040, and antifungal activity was assessed according to the European standard 1650 by enumerating colony‐forming units after a 24‐h incubation period.

**Results:**

Four major phytochemicals — alpha‐terpineol, linalool, myrcene, and verbenone — were identified as key bioactive compounds. These exhibited favorable pharmacokinetic properties and predicted interactions with targets related to inflammation, oxidative stress, and microbial virulence. *In vitro*, the ethanolic extract of *P. lentiscus* achieved ≥ 5 log reductions in both bacterial and fungal strains, demonstrating potent antimicrobial activity comparable to chlorhexidine. The hydroalcoholic extract showed moderate yet significant reductions.

**Conclusion:**

The integrative in silico and in vitro analyses indicate that *P. lentiscus* exerts antimicrobial effects through multiple complementary mechanisms, including membrane disruption, inhibition of virulence and biofilm formation, and modulation of host inflammatory pathways. These findings provide a mechanistic framework supporting the use of *P. lentiscus* as a natural adjunct for managing periodontal infections and warrant further preclinical and clinical evaluation.

## 1. Introduction

Periodontitis is a persistent inflammatory condition impacting the periodontal ligaments and alveolar bone [1]. The accumulation of plaque and subsequent biofilm formation, resulting from the colonization of diverse microorganisms, is a primary etiological factor in the pathogenesis of periodontitis [1]. The first step in the management of periodontal disease is control of the microbial load [2]. Phytomedicine, or herbal medicine, refers to the utilization of plant‐derived active ingredients that are widely accepted to provide preventive and therapeutic benefits in various medical fields [3]. Phytomedicines have been shown to exhibit antimicrobial, antioxidant, and anti‐inflammatory properties, thus playing a significant role in managing periodontal diseases [4, 5].

Network pharmacology is an emerging discipline that integrates network biology and polypharmacology [6]. This in silico approach to drug design has shifted the paradigm from a one‐target, one‐drug mode to a multi‐target, multi‐component therapeutics mode [7]. Novel network pharmacology approaches serve as valuable tools in evidence‐based Ayurveda for the discovery of bioactive compounds and the elucidation of the mechanisms of action of herbal formulas.

Mastic gum is a white, semi‐transparent, natural, aromatic resin obtained from the mastic tree (*Pistacia lentiscus* var. Chia) [8]. The chemical composition of mastic gum has been studied by several researchers and has been found to be complex, comprising ~120 different compounds [6]. Many of these compounds exhibit various biological activities, including antimicrobial [9], antioxidant, anti‐inflammatory [10], hypolipidemic [11], anti‐diabetic, and anticancer [12] properties.

The European Committee for Standardization (CEN) created standards for evaluating the antibacterial (PN‐EN 1040) and antifungal (EN 1650) activities of various products to indicate whether a product exhibits antimicrobial effects and meets recognized efficacy benchmarks [13]. Although the antimicrobial efficacy of mastic gum has been assessed against various bacteria [14, 15], its efficacy is yet to be assessed using standard antimicrobial guidelines such as PN‐EN 1040 and EN 1650.

In the present study, we used a network pharmacology approach to explore potential mechanisms of action of *P. lentiscus* with regard to its antibacterial properties and the molecular mechanisms underlying the antibacterial and antifungal effects. In addition, we carried out in vitro analyses of the antibacterial and antifungal efficacies of the hydroalcohol and ethanolic extracts of *P. lentiscus* in accordance with the Polskie Normy‐European Norms 1040 (PN‐EN1040) and European Norms 1650 (EN 1650) standard bacterial and fungal strains.

## 2. Material and Methods

### 2.1. Network Pharmacology

#### 2.1.1. Data Mining

The bioactive compounds in *P. lentiscus* var. Chia (mastic gum) was obtained from relevant literature and online databases (Supporting Information 1: Table S1). The data were primarily sourced from the Indian Medicinal Plants, Phytochemistry, and Therapeutics (IMPPAT) database, which is the largest database of Indian medicinal plant phytochemicals to date. A comprehensive list of the physicochemical properties of the compounds was compiled from the available literature and chemical databases, including PubChem. Protein targets for the individual compounds were gathered using tools such as Swiss Target Prediction and SuperPred.

#### 2.1.2. Virtual Screening

Here, we aimed to identify compounds with antibacterial activity. Not all the numerous bioactive compounds present in medicinal plants possess the required drug characteristics. To identify compounds with the potential to act as antibacterial agents, we eliminated the least essential compounds based on data obtained from research articles and screened them based on their absorption, distribution, metabolism, and excretion properties (SwissADME).

The list of compounds was further narrowed down by analyzing the drug‐likeness data, which were derived using Lipinski’s rule of five, a set of principles used in drug discovery to evaluate the drug‐likeness and pharmacokinetic properties of potential compounds, using the following criteria: molecular weight (< 500 Da), lipophilicity (LogP <5), hydrogen bond donors (<5), and hydrogen bond acceptors (<10). Eleven compounds with antibacterial properties were identified: alpha‐terpineol, linanool, alpha‐pinene, isomasticadienolic acid, masticadienolic acid, myrcene, oleanolic acid, shikimic acid, quinic acid, and quercetin.

#### 2.1.3. Antibacterial Compounds

The molecular structures of the 11 compounds were obtained from the National Center for Biotechnology Information (NCBI) PubChem database, and their 3D structures of the identified compounds were developed using Marvin Sketch software. Energy minimization was performed using the Avogadro software. The most stable 3‐dimensional conformation of the compound was obtained and saved as a PDB file for further use.

The list of compounds was further refined based on the solubility and absorption rates of the individual compounds, which predicted their potential to act as drugs in the biological environment. Compounds with high solubility were included because they can be used as drugs through different administration routes, such as oral and topical. Based on these results, four antibacterial compounds were identified.

The metabolic behavior of the compounds was predicted using the ADME SIB portal and ADMETSAR 2.0. Toxicity assessments were performed to analyze and estimate toxicity levels. Finally, the bioactivities of the compounds were predicted using the Prediction of Activity Spectra for Substances (Pass Online) server.

**Figure 1:**
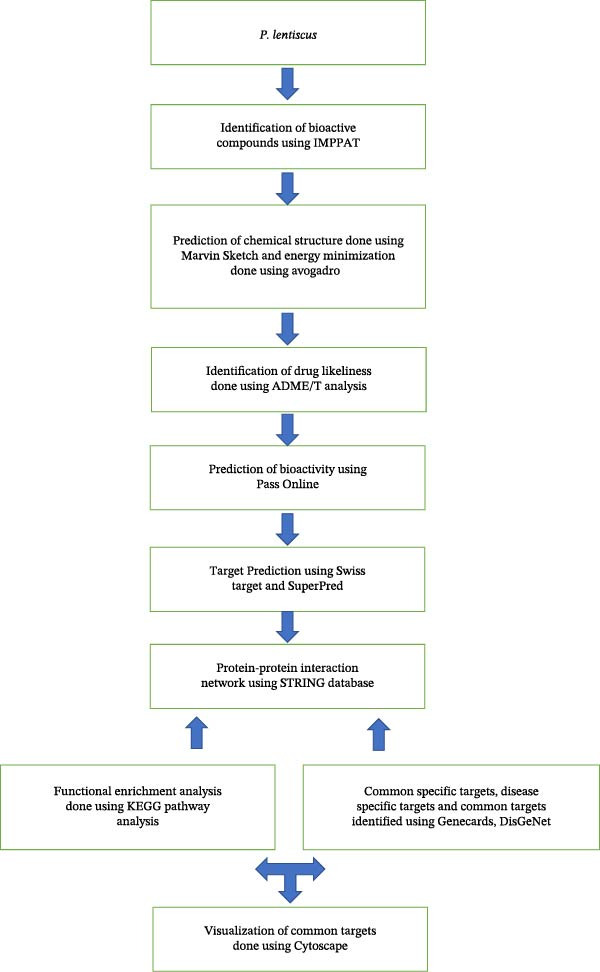
Methodology for in silico analysis.

The protein targets for the various compounds, along with their gene IDs, family details, associated biological processes, and molecular functions, were mined and tabulated. Kyoto Encyclopedia of Genes and Genomes (KEGG) pathway analysis was carried out, and a protein–protein interaction network was retrieved from the Search Tool for the Retrieval of Interacting Gene/Proteins (STRING) database for compound‐specific targets predicted using Swiss Target Prediction and SuperPred. Disease‐specific targets were also obtained from databases including Genecards, Online Mendelian Inheritance in Man (OMIM), and DisGeNet (Figure 1).

## 3. In Vitro Analysis

The study protocol was approved by the Institutional Ethics Committee (CSP/22/OCT/117/523).

### 3.1. Sample Preparation

This study included both test and control groups. Positive and negative controls were used to validate the results. The positive control used was chlorhexidine. Two negative controls were included: Control A: 0.125 mL of the bacterial‐fungal suspension in 1 mL of Brain Heart Infusion (BHI) medium. Control B: 0.125 mL of the bacterial‐fungal suspension mixed with 1 mL of Dey‐Engley neutralizer and 0.125 mL of artificial saliva. Commercially available mastic gum was procured and validated by the Siddha Central Research Institute, Chennai. Hydroalcoholic and ethanolic extracts were obtained from the resin using Soxhlet extraction followed by the rotavapor concentration method. The test products were divided into two groups, and their antibacterial and antifungal activities were assessed.

### 3.2. Antibacterial and Antifungal Activity

**Figure 2:**
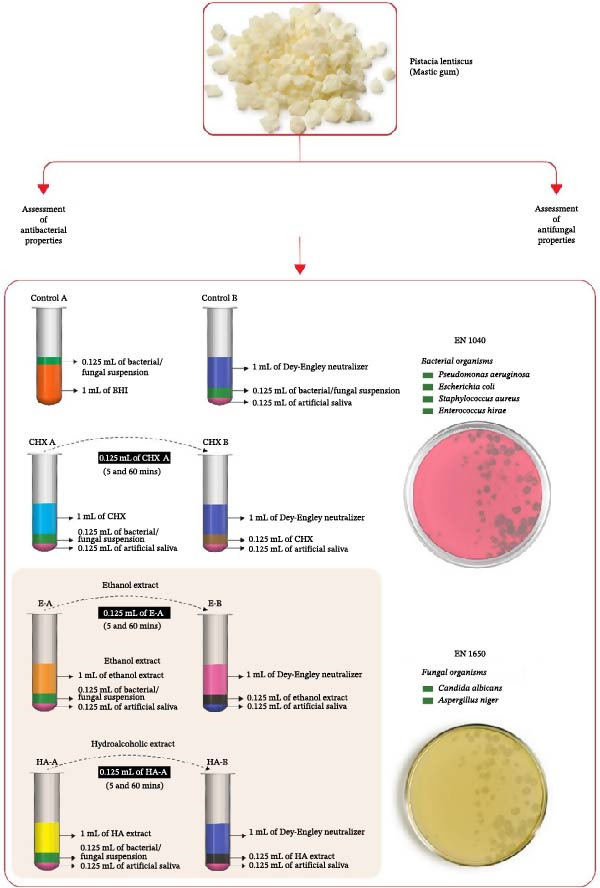
Methodology for in vitro analysis.

Bactericidal activity was assessed in accordance with the PN‐EN 1040 standard, and antifungal activity was assessed in accordance with EN 1650. For a product to be considered antibacterial, a minimum 5 log reduction in the number of live bacteria is required within a contact time of 60 min or less. A minimum log reduction of 4 or greater was presumed to show that products possess antifungal properties. *Staphylococcus aureus* (ATCC 6538), *Pseudomonas aeruginosa* (ATCC 15,442), *Escherichia coli* (ATCC 10,538), and *Enterococcus hirae* (ATCC 10,541) were the bacteria used. *Candida albicans* (ATCC 90,028) and *Aspergillus niger* (ATCC‐6275) were the fungal strains used. The densities of the bacterial and fungal suspensions were adjusted to 1.5–5 × 10^8^ colony‐forming units (CFU)/mL. Two contact times (5 and 60 min) were used for the analysis. At the end of the contact period, bactericidal and fungal activities were immediately terminated using Dey–Engley neutralizing broth [16]. Briefly, 1 mL of the test product (hydroalcohol and ethanol extracts of mastic gum or chlorhexidine) was combined with 0.125 mL of artificial saliva and 0.125 mL of the bacterial/fungal test suspension. At the end of the contact time (5 or 60 min ± 10 s), a 0.125 mL sample of the test mixture was added to a tube containing 1 mL of neutralizer mixed with 0.125 mL of artificial saliva. An aliquot (0.01 mL) of the neutralized test combination was immediately removed after 5 min ± 10 s of neutralization time and inoculated onto blood agar plates. This step was performed in triplicate. Finally, the number of CFUs was counted after 24 h of incubation at 37°C (FIGURE 2).

### 3.3. Calculation of Bacterial and Mycotic Counts

For the final assay (Na) in the preliminary assays (A, B, C, and Nv) and the test suspension (N), the bacterial and mycotic counts were calculated as follows:

CFU/mL=Cn×V×d,

where *C* is the total number of colonies counted in all counted dishes, *n* is the number of counted dishes, *V* is the volume used, and *d* is the dilution factor corresponding to the relevant dilution. The number of colonies in each dish was counted.

## 4. Statistical Analysis

The Kruskal–Wallis test was used to compare between groups, and the Wilcoxon signed‐rank test was used to compare within groups using SPSS software. Statistical significance was set at *p*  < 0.05.

## 5. Results

### 5.1. Network Pharmacology

Compounds that did not satisfy the Lipinski rule of five were deemed unsuitable for use as drugs and would require modifications to be used as drugs. Compounds with high solubility were included. The list of 11 compounds to be included in the network pharmacology analysis was narrowed down to four (alpha‐terpineol, linalool, myrcene, and verbenone; Table 1). The gene targets associated with the four bioactive compounds of *P. lentiscus* were predicted using the SWISS TARGET database. The specific targets were predicted and tabulated (Supporting Information 1: Table S2). The protein–protein interaction network was retrieved from the STRING database for compound‐specific targets (Supporting Information 2: Figure A). Gene Ontology (GO) annotation was performed to reveal biological processes, molecular functions, and cellular components associated with the target genes. KEGG pathway analysis was also performed (Supporting Information 2: Figure B–E). Antimicrobial activity is often mediated not only by direct microbicidal effects but also through modulation of host‐related pathways, such as inflammation, oxidative stress, and immune signaling, which can influence microbial survival and colonization. Identifying compound–host protein interactions helps anticipate potential off‐target effects and safety considerations, thereby strengthening the translational value of the identified compounds. Therefore, the STRING and GO analyses complement the in vitro antimicrobial assays by providing a systems‐level perspective on how these phytochemicals may exert both direct antimicrobial and indirect host‐modulatory effects.

**Table 1:** Pharmacokinetic properties of bioactive compounds of *Pistachia lentiscus* (ADME/T prediction).

Prediction	Alpha‐pinene	Alpha‐terpineol	Isomasticadienolic acid	Linalool	Masticadienolic acid	Myrcene	Oleanolic acid	Quercetin	Quinic acid	Shikimic acid	Verbenone
**Absorption**											
Human intestinal absorption	100	100	96.066441	100	96.066	100	95.996	63.485215	20.2869	43.066	100
CaCo_2_ permeability	23.6322	50.8083	22.2664	29.355	22.2813	23.6306	21.8872	3.4129	8.272	14.229	32.5318
Bioavailability score	0.55	0.55	0.85	0.55	0.85	0.55	0.85	0.55	0.56	0.56	0.55
Solubility	Soluble	Soluble	Poorly soluble	Soluble	Poorly soluble	Soluble	Poorly soluble	Soluble	Highly soluble	Highly soluble	Soluble
Lipinski rule of 5	Yes, 1 violation	Yes, 0 violation	Yes, 1 violation	Yes, 0 violation	Yes, 1 violation	Yes, 0 violation	Yes, 1 violation	Yes, 0 violation	Yes, 0 violation	Yes, 0 violation	Yes, 0 violation
**Distribution**											
P‐glycoprotein inhibitor	Yes	No	Yes	No	Yes	Yes	Yes	No	No	No	Yes
Blood brain barrier penetration	Yes	Yes	No	Yes	No	Yes	No	No	No	No	Yes
Plasma protein binding	100	23.416307	100	100	100	100	100	93.236	6.595	23.792	100
**Metabolism**											
CYP 2C19 inhibition	No	Yes	No	Yes	No	Yes	Yes	Yes	No	Yes	No
CYP 2C9 inhibition	Yes	Yes	Yes	Yes	Yes	Yes	Yes	Yes	Yes	Yes	Yes
**Excretion**											
Clearance Rate (ml/min/kg)	15.022 High	8.942 Moderate	7.250 Moderate	9.738 Moderate	6.502 Moderate	13.108 Moderate	2.248 Low	8.284 Moderate	1.556 Low	3.591 Low	6.956 Moderate
**Toxicity**											
Hepatotoxicity	Inactive	Inactive	Inactive	Inactive	Inactive	Inactive	Active	Inactive	Inactive	Inactive	Inactive
Carcinogenicity	Inactive	Inactive	Active	Inactive	Active	Inactive	Active	Active	Inactive	Inactive	Inactive
Immunotoxicity	Inactive	Inactive	Active	Inactive	Active	Inactive	Active	Inactive	Inactive	Inactive	Inactive
Mutagenicity	Inactive	Inactive	Inactive	Inactive	Inactive	Inactive	Inactive	Active	Inactive	Inactive	Inactive
Cytotoxicity	Inactive	Inactive	Inactive	Inactive	Inactive	Inactive	Inactive	Inactive	Inactive	Inactive	Inactive

Based on the confidence values obtained, the antibacterial activity of the four compounds (alpha‐terpineol, linalool, myrcene, and verbenone) was assessed for various microorganisms. A confidence score of 0.7 and above was considered to indicate good antibacterial activity, while a confidence score less than 0.3 was considered to indicate poor antibacterial activity. These are the data compiled from previously published literature and validated using open‐access databases, including IMPPAT (https://cb.imsc.res.in/imppat/) [17], PubChem (https://pubchem.ncbi.nlm.nih.gov/) [18], and Swiss Target Prediction (https://www.swisstargetprediction.ch/) [19] (Table 2). All four compounds exhibited antibacterial activity against *Prevotella intermedia* and *Porphyromonas gingivalis*, confirming their potential for use in the treatment of periodontitis. Compound‐ and disease‐specific targets were assessed, common targets were elucidated, and networks were constructed using Cytoscape (Supporting Information 2: Figure F).

**Table 2:** Antibacterial activity (data compiled from previously published literatures) [17–19].

Microorganisms	Alpha‐terpineol	Linanool	Myrcene	Verbenone
*Prevotella melaninogenica*	0.6222	—	—	0.2227
*Prevotella intermedia*	**0.5889**	**0.4129**	**0.4891**	**0.27**
*Streptococcus mutans*	0.5538	0.2838	0.3391	—
Resistant *Staphylococcus simulans*	0.5505	0.3305	—	0.4021
*Prevotella oralis*	0.5298	—	—	—
*Mycobacterium mageritense*	0.4656	—	—	—
*Salmonella enteritidis*	0.4483	—	—	—
*Staphylococcus sciuri*	0.4393	0.225	—	0.293
*Micrococcus luteus*	0.3902	—	—	—
*Staphylococcus simulans*	0.3781	0.5192	0.6409	0.2197
*Corynebacterium jeikeium*	0.3531	—	—	—
*Porphromonas gingivalis*	**0.3392**	**0.3069**	**0.3184**	**0.2189**
*Staphylococcus lugdunensis*	—	0.3922	0.3985	—
*Lactobacillus planatarum*	—	0.392	0.4303	—
*Streptococcus sanguinis*	—	0.3302	0.3677	—
*Listeria monocytogenes*	—	0.3024	0.4306	—
*Mycobacterium bovis*	—	—	0.3346	0.2722

*Note:* Bold values emphasise the antibacterial activity against putative periodontal pathogens.

### 5.2. In Vitro Analysis

The present study showed that mastic gum extracts reduced bacterial and fungal counts across all the tested strains. Furthermore, chlorhexidine mouthwash showed a ≥5 log reduction in both the bacterial and fungal strains tested at both time points. The ethanol extract of mastic gum resin also showed ≥5 log reduction in both the bacterial and fungal strains at both time points. The hydroalcohol extract showed ≥5 log reduction for *P. aueroginosa*, while it showed ≥4 log reduction for other bacterial and fungal strains. A 2 log reduction was observed for *E. hirae* was treated with the hydroalcohol extract at both time points (Table 3) (Supporting Information 2: Figures G–L). The intergroup differences between all the bacterial and fungal strains were statistically significant, while the intragroup differences in terms of log reduction at the 5 and 60 min time points were not statistically significant (Table 4).

**Table 3:** Antibacterial, antifungal activity of chlorhexidine, hydroalcohol and ethanol.

EN Organisms	PN‐EN 1040								EN‐1650			
	*Pseudomonas aeuroginosa*		*Escherichia coli*		*Staphylococcus aureus*		*Enterococcus* *hirae*		*Candida albicans*		*Aspergillus niger*	
Contact time (log reduction) (5 min)	5	60	5	60	5	60	5	60	5	60	5	60
CHX	>5	>5	>5	>5	>5	>5	>5	>5	>5	>5	>5	>5
Ethanol	>5	>5	>5	>5	>5	>5	>5	>5	>5	>5	>5	>5
Hydroalcohol	>5	>5	4	4	4	>5	2	3	4	5	>5	>5

**Table 4:** Comparison of antibacterial and antifungal activity between groups.

EN organisms	PN‐EN 1040												EN‐1650					
	*Pseudomonas aeuroginosa*			*Escherichia coli*			*Staphylococcus aureus*			*Enterococcus* *hirae*			*Candida albicans*			*Aspergillus niger*		
	Mean rank	Chi‐square	*p*‐value	Mean rank	Chi‐square	*p*‐value	Mean rank	Chi‐square	*p*‐value	Mean rank	Chi‐square	*p*‐value	Mean rank	Chi‐square	*p*‐value	Mean rank	Chi‐square	*p*‐value
Control A	11.00	11.00	0.012	11.00	10.822	0.013	11.00	10.822	0.013	11.00	10.583	0.014	11.00	10.68	0.014	11.00	11.00	0.012
Control B	11.00	11.00	0.012	11.00	10.822	0.013	11.00	10.822	0.013	11.00	10.822	0.013	11.00	10.822	0.013	11.00	11.00	0.012
CHX 5 min	5.00	11.00	0.012	3.50	10.822	0.013	3.50	10.822	0.013	2.00	10.583	0.014	2.00	10.68	0.014	5.00	11.00	0.012
CHX 60 min	5.00	11.00	0.012	3.50	10.822	0.013	3.50	10.822	0.013	3.50	10.822	0.013	3.50	10.822	0.013	5.00	11.00	0.012
Mastic gum‐ethanol 5 min	5.00	11.00	0.012	3.50	10.822	0.013	3.50	10.822	0.013	8.00	10.583	0.014	8.00	10.68	0.014	5.00	11.00	0.012
Mastic gum‐ethanol 60 min	5.00	11.00	0.012	3.50	10.822	0.013	3.50	10.822	0.013	3.50	10.822	0.013	3.50	10.822	0.013	5.00	11.00	0.012
Mastic gum‐hydroalcohol 5 min	5.00	11.00	0.012	8.00	10.822	0.013	8.00	10.822	0.013	5.00	10.583	0.014	5.00	10.68	0.014	5.00	11.00	0.012
Mastic gum‐hydroalcohol 60 min	5.00	11.00	0.012	8.00	10.822	0.013	8.00	10.822	0.013	8.00	10.822	0.013	8.00	10.822	0.013	5.00	11.00	0.012

## 6. Discussion

ADME/T predictions were performed on eleven phytochemicals. Based on favorable drug‐likeness and predicted biological activities, alpha‐terpineol, linalool, myrcene, and verbenone were selected for further investigation.

### 6.1. Pharmacokinetic Evaluation

The ADME analysis indicated high human intestinal absorption and acceptable bioavailability scores for all four compounds, which supports their suitability as orally administered drugs. These findings align with previous studies that have reported high permeability and bioavailability for terpenoid compounds [20]. Furthermore, all shortlisted compounds complied with Lipinski’s Rule of Five, which predicts good oral bioavailability [21]. While Lipinski’s rule of five provides a widely accepted foundation for assessing drug‐likeness, it is increasingly recognized that many clinically approved drugs, particularly natural products and their derivatives, do not conform to these parameters. Such molecules may exert pharmacological effects through non‐traditional mechanisms, transporter‐mediated uptake, or localized activity that bypasses classical absorption and distribution constraints. Therefore, deviations from Lipinski’s rule should not preclude the therapeutic potential of phytochemicals such as those identified in *P. lentiscus*. This perspective is supported by Doak et al. [22], who demonstrated that a substantial proportion of modern oral drugs violate one or more of Lipinski’s criteria yet retain potent and selective bioactivity.

Alpha‐terpineol, linalool, and verbenone have been shown to permeate the blood‐brain barrier, indicating potential effects on the central nervous system. This observation is consistent with the work of Raut and Karuppayil, who noted the neuroactive properties of essential oil constituents like linalool [23]. The high plasma protein binding (>90%) observed for all compounds is a double‐edged sword; while it can enhance drug stability in plasma, it may reduce free drug concentration [24].

All four compounds were predicted to inhibit cytochrome P450 enzymes (especially CYP2C9), raising concerns about possible drug–drug interactions [25]. The four leading compounds were found to be non‐mutagenic and non‐cytotoxic in toxicity studies. On the other hand, oleanolic acid and quercetin, despite their pharmacological activity, were identified as potentially hepatotoxic and carcinogenic and were therefore not considered for further study.

### 6.2. Target Prediction and Network Pharmacology

Target prediction using the SWISS tool identified diverse protein interactions for the shortlisted compounds. Notably, alpha‐terpineol and verbenone demonstrated extensive molecular interactions with key proteins such as acetylcholinesterase, cannabinoid receptors, and cytochrome P450 isoforms. These findings align with earlier studies highlighting the pleiotropic effects of terpenes in modulating inflammatory and neuroactive pathways [26].

Network pharmacology analysis revealed a strong overlap between compound‐specific and disease‐related targets. Alpha‐terpineol had the highest number of shared targets (12), followed by verbenone (10), linalool (9), and myrcene (6), highlighting their polypharmacological potential. These results underscore the growing interest in network pharmacology for uncovering multi‐target therapeutic agents from plant sources [27].

### 6.3. Antibacterial Activity

Predictions of antibacterial activity indicated that the four compounds selected demonstrated moderate to high activity against a range of oral and pathogenic bacteria. Specifically, all four compounds exhibited effectiveness against *P. intermedia* and *P. gingivalis*, key contributors to periodontitis. This aligns with existing literature that documents the antimicrobial characteristics of linalool and alpha‐terpineol against both gram‐negative and gram‐positive bacteria [28, 29].

Alpha‐terpineol demonstrated a broad spectrum of antibacterial activity, exhibiting notable efficacy against *Streptococcus mutans*, *Staphylococcus simulans*, *and Bacillus cereus*, as indicated by confidence scores exceeding 0.5. Linalool and myrcene also displayed antibacterial effects against *Listeria monocytogenes and Lactobacillus plantarum*, suggesting their possible application as natural antimicrobial agents.

European Standards developed by CEN provide benchmarks for assessing the antibacterial and antifungal properties of different products. These standards stipulate that effectiveness against standard‐resistant strains signifies antimicrobial activity, meeting established efficacy criteria. The minimum duration of contact between a product and the oral cavity is 5 min, allowing for immediate effect analysis. The product may remain in contact with the oral cavity for an extended period, up to 60 min. Therefore, the immediate effect exerted by the product can be analyzed at this point [30]. The product can remain in contact with the oral cavity for a longer duration, extending to a maximum of 60 min.

This study sought to assess the antibacterial and antifungal effects of ethanol and hydroalcoholic extracts of mastic gum, comparing them against established PN‐EN 1040 and EN 1650 standards. The antibacterial activity of mastic gum components was evaluated against resistant strains of *S. aureus*, *P. aeruginosa*, *E. coli*, *and E. hirae*, while antifungal activity was tested against pure strains of *C. albicans and A. niger*. Results indicated that chlorhexidine mouthwash significantly inhibited specific bacterial and fungal strains at both evaluated time points. The ethanol extract of mastic gum notably reduced colony numbers for both bacterial and fungal strains at both time points. The hydroalcoholic extract demonstrated greater than 4 log reductions in bacterial and fungal colony numbers at both time points, except for *E. hirae*, which showed only a 2 log reduction at 5 min and a 3 log reduction at 60 min. Intergroup differences between all bacterial and fungal strains were statistically significant, whereas intragroup differences in log reduction of colony numbers at 5 and 60 min were not statistically significant. The ethanol extract of mastic gum resin exhibited superior antimicrobial efficacy compared to the hydroalcoholic extract, although this difference was not statistically significant, underscoring the potential antimicrobial capabilities of mastic gum resin, with the ethanol extract demonstrating greater promise for antibacterial and antifungal applications.

The antibacterial activity of *P. lentiscus* appears to result from multiple complementary mechanisms supported by in silico analyses. These compounds interact with molecular targets involved with bacterial survival and host‐pathogen dynamics. At the microbial level, alpha‐terpineol and linalool can disrupt bacterial cell walls by altering their permeability and fluidity, resulting in leakage and cell death [31–33]. This is particularly specific to gram‐negative microorganisms such as *P. gingivalis* and *P. intermedia*. Target prediction and docking further suggest inhibition of bacterial virulence factors and enzymes linked to oxidative stress defense and biofilm formation, thereby impairing bacterial adhesion and colonization [34–36].

In addition, network pharmacology analysis revealed modulation of host pathways, including inflammatory and oxidative stress signaling suggesting that *P. lentiscus* compounds may also mitigate the exaggerated inflammatory response [28, 37, 38]. This host‐modulatory activity is consistent with the previous reports showing that mastic gum components reduce cytokine release, reactive oxygen species, and inflammation in oral epithelium [39–41]. Thus the in vitro activity is found to be arising from a combination of membrane disruption, virulence and biofilm inhibition, and host response modulation, together maintaining microbial‐host homeostasis in the periodontal environment [42, 43]. The limitation of the study is that antimicrobial assessment was not specifically performed against putative periodontal pathogens under conditions mimicking the oral environment. Additionally, it should be noted that standardized international protocols for evaluating oral disinfecting agents or natural compounds targeting periodontal pathogens are currently lacking, which constrains direct comparison across studies.

## 7. Conclusion

This study highlights *P. lentiscus* as a promising source of antimicrobial agents by integrating in silico predictions with standardized in vitro assays. Four key phytochemicals — alpha‐terpineol, linalool, myrcene, and verbenone — demonstrated propitious pharmacokinetic properties, multi‐target potential, and predicted activity against periodontitis‐associated pathogens. Ethanol extracts of mastic gum achieved bactericidal and fungicidal effects consistent with European Standards, confirming their broad‐spectrum efficacy. Collectively, these findings provide a strong basis for advancing *P. lentiscus* derivatives into further preclinical and clinical studies for combating periodontal infections.

## Funding

This research received no specific grants from any funding agency in the public, commercial, or not‐for‐profit sectors.

## Conflicts of Interest

The authors declare no conflicts of interest.

## Supporting Information

Additional supporting information can be found online in the Supporting Information section.

## Supporting information


**Supporting Information 1** Table S1: Bioactive compounds of *Pistacia lentiscus*. Table S2: Target prediction of bioactive compounds

**Supporting Information 2** Figure A: Protein–protein interaction network retrieved from STRING database for the compound‐specific targets‐alpha‐terpineol, linalool, myrcene, verbenone. Figure B: Functional enrichment analysis: alpha‐terpineol A. Biological process B. Molecular process C. Cellular process D. KEGG pathway analysis. Figure C: Functional enrichment analysis: linalool A. Biological process B. Molecular process C. Cellular process D. KEGG pathway analysis. Figure D: Functional enrichment analysis: verbenone A. Biological process B. Molecular process C. Cellular process D. KEGG pathway analysis. Figure E: Functional enrichment analysis: myrcene ‐ KEGG pathway analysis. Figure F: cytoscape‐verbenone, myrcene, linalool. Figure G: CFU counts on agar *plates-Pseudomonas aeuroginosa*. Figure H: CFU counts on agar plates‐*Escherichia coli*. Figure I: CFU counts on agar plates‐*Staphylococcus aureus*. Figure J: CFU counts on agar plates‐*Enterococcus hirae*. Figure K: CFU counts on SDA plates‐*Aspergillus niger*. Figure L: CFU counts on SDA plates‐*Candida albicans*.

## Data Availability

The data that support the findings of this study are available from the corresponding author upon reasonable request.
