# Supplementary material for: In Vitro Evaluation and Network Pharmacology Analysis of the Antimicrobial Activity of Pistacia lentiscus
Source: Int J Dent. 2026 Jan 20;2026:6981413. doi: 10.1155/ijod/6981413 (PMC12817137; doi:10.1155/ijod/6981413)
Supplement: Supplementary file 1 — Supporting Information 1 Table S1: Bioactive compounds of Pistacia lentiscus. Table S2: Target prediction of bioactive compounds [file IJOD-2026-6981413-s001.docx]

**SUPPLEMETARY FILES-TABLE**

BIOACTIVE COMPOUNDS OF PISTACIA LENTISCUS VAR CHIA

| **S.No** | **Compound name** | **Canonical SMILES** | **Pubchem ID** |
| --- | --- | --- | --- |
| 1 | 1,4-Cineole | CC(C12CCC(O2)(CC1)C)C |  |
| 2 | 4-Isopropylbenzaldehyde | O=Cc1ccc(cc1)C(C)C |  |
| 3 | Cadalene | Cc1ccc2c(c1)c(ccc2C)C(C)C |  |
| 4 | (-)-beta-Bourbonene | CC([C@@H]1CC[C@@]2([C@H]1[C@H]1C(=C)CC[C@@H]21)C)C |  |
| 5 | (+)-gamma-Gurjunene | C[C@@H]1CC[C@H]2C1=C[C@@H](CC[C@H]2C)C(=C)C |  |
| 6 | (E)-2,6-Dimethylocta-5,7-dien-4-one | C=C/C(=C/C(=O)CC(C)C)/C |  |
| 7 | 1,3-p-Menthadien-7-al | O=CC1=CC=C(CC1)C(C)C |  |
| 8 | 1-Hexanol | CCCCCCO |  |
| 9 | 1-Methyl-4-(prop-1-en-2-yl)benzene | Cc1ccc(cc1)C(=C)C |  |
| 10 | 1-Octen-3-OL | CCCCCC(C=C)O |  |
| 11 | 2-(4-Methylphenyl)propan-2-ol | Cc1ccc(cc1)C(O)(C)C |  |
| 12 | 2,4,5-Trimethylbenzaldehyde | O=Cc1cc(C)c(cc1C)C |  |
| 13 | 2,4-Decadienal | CCCCC/C=C/C=C/C=O |  |
| 14 | 2-Nonanol | CCCCCCCC(O)C |  |
| 15 | ALPHA-PINENE | CC1=CCC2CC1C2(C)C | 6654 |
| 16 | AUCUBIN | C1=COC(C2C1C(C=C2CO)O)OC3C(C(C(C(O3)CO)O)O)O | 91458 |
| 17 | Benzyl isovalerate | CC(CC(=O)OCc1ccccc1)C |  |
| 18 | beta-Bisabolene | CC(=CCCC(=C)[C@H]1CCC(=CC1)C)C |  |
| 19 | BETA-CARYOPHYLLENE | CC1=CCCC(=C)C2CC(C2CC1)(C)C | 5281515 |
| 20 | beta-Cubebene | CC([C@@H]1CC[C@H]([C@]23[C@H]1[C@H]2C(=C)CC3)C)C |  |
| 21 | BETA-PINENE | CC1(C2CCC(=C)C1C2)C | 14896 |
| 22 | beta-Sinensal | C=CC(=C)CC/C=C(/CC/C=C(/C=O)C)C |  |
| 23 | Bicyclogermacrene | C/C/1=CCC/C(=C/[C@H]2[C@@H](CC1)C2(C)C)/C |  |
| 24 | CHOLINE | C[N+](C)(C)CCO | 305 |
| 25 | Cubebol | CC([C@@H]1CC[C@H]([C@]23[C@H]1[C@H]2[C@@](C)(O)CC3)C)C |  |
| 26 | Cubenene | C12C3C4C2=C2C1C3=C42 |  |
| 27 | CYCLOARTENOL | CC(CCC=C(C)C)C1CCC2(C1(CCC34C2CCC5C3(C4)CCC(C5(C)C)O)C)C | 92110 |
| 28 | Daucene | CC1=CCC2(C(=C(CC2)C(C)C)CC1)C |  |
| 29 | Elemicin | C=CCc1cc(OC)c(c(c1)OC)OC |  |
| 30 | gamma-Terpinene | CC1=CCC(=CC1)C(C)C |  |
| 31 | Isopentyl benzoate | CC(CCOC(=O)c1ccccc1)C |  |
| 32 | KAEMPFEROL | C1=CC(=CC=C1C2=C(C(=O)C3=C(C=C(C=C3O2)O)O)O)O | 5280863 |
| 33 | LINALOOL | CC(=CCCC(C)(C=C)O)C | 6549 |
| 34 | Linalyl propionate | CCC(=O)OC(CCC=C(C)C)(C=C)C |  |
| 35 | Longifolene | C=C1C2CCC3C1(C)CCCC(C23)(C)C |  |
| 36 | LUPEOL | CC(=C)C1CCC2(C1C3CCC4C5(CCC(C(C5CCC4(C3(CC2)C)C)(C)C)O)C)C | 259846 |
| 37 | MYRCENE | CC(=CCCC(=C)C=C)C | 31253 |
| 38 | Myrcene | C=CC(=C)CCC=C(C)C |  |
| 39 | MYRICETIN | C1=C(C=C(C(=C1O)O)O)C2=C(C(=O)C3=C(C=C(C=C3O2)O)O)O | 5281672 |
| 40 | Myristic acid | CCCCCCCCCCCCCC(=O)O |  |
| 41 | Myrtenal | O=CC1=CCC2CC1C2(C)C |  |
| 42 | Myrtenol | OCC1=CCC2CC1C2(C)C |  |
| 43 | O-Cymene | CC(c1ccccc1C)C |  |
| 44 | OLEANOLIC-ACID | CC1(CCC2(CCC3(C(=CCC4C3(CCC5C4(CCC(C5(C)C)O)C)C)C2C1)C)C(=O)O)C | 10494 |
| 45 | p-Cymene | Cc1ccc(cc1)C(C)C |  |
| 46 | Pinocarvone | C=C1C(=O)CC2CC1C2(C)C |  |
| 47 | QUERCETIN | C1=CC(=C(C=C1C2=C(C(=O)C3=C(C=C(C=C3O2)O)O)O)O)O | 5280343 |
| 48 | QUINIC-ACID | C1C(C(C(CC1(C(=O)O)O)O)O)O | 6508 |
| 49 | RHINANTHIN | C1=COC(C2C1C(C=C2CO)O)OC3C(C(C(C(O3)CO)O)O)O | 348157 |
| 50 | SHIKIMIC-ACID | C1C(C(C(C=C1C(=O)O)O)O)O | 8742 |
| 51 | Spathulenol | C=C1CC[C@@H]2[C@H]([C@H]3[C@H]1CC[C@]3(C)O)C2(C)C |  |
| 52 | TIRUCALLOL | CC(CCC=C(C)C)C1CCC2(C1(CCC3=C2CCC4C3(CCC(C4(C)C)O)C)C)C | 101257 |
| 53 | Tricyclene | CC12C3C1CC(C2(C)C)C3 |  |
| 54 | Verbenone | CC1=CC(=O)C2CC1C2(C)C |  |
| 55 | Dammaradienone | CC(=CCCC(=C)C1CCC2(C1CCC3C2(CCC4C3(CCC(=O)C4(C)C)C)C)C)C |  |
| 56 | Limonene |  |  |
| 57 | Alpha Terpineol | CC1=CCC(CC1)C(C)(C)O |  |
| 58 | Isomasticadienolic acid | CC(CCC=C(C)C(=O)O)C1CCC2(C1(CCC3=C2CCC4C3(CCC(C4(C)C)O)C)C)C |  |
| 59 | Masticadienolic acid |  |  |

**TARGET PREDICTION:**

The gene targets associated with P.lentiscus were predicted through the SWISS TARGET prediction for 4 bioactive compounds.

| **BIOACTIVE COMPOUNDS** | **PROTEIN TARGETS** |
| --- | --- |
| ALPHA-TERPINEOL | 11-beta-hydroxysteroid dehydrogenase 1, Acetylcholinesterase ,Acyl-CoA desaturase ,Adrenergic receptor alpha-2 ,Aldo-keto reductase family 1 member B10 ,Androgen Receptor, Bloom syndrome protein ,Butyrylcholinesterase ,Cannabinoid CB2 receptor ,Carbonic anhydrase I ,Carbonic anhydrase II ,Carbonic anhydrase IV ,Cathepsin D ,CD81 antigen , Cytochrome P450 17A1 ,Cytochrome P450 19A1,Cytochrome P450 2C19 ,Cytochrome P450 51 ,Dipeptidyl peptidase VIII ,DNA topoisomerase II alpha ,DNA-(apurinic or apyrimidinic site) lyase ,Dopamine D2 receptor (by homology) ,Dopamine transporter ,Estrogen receptor alpha , Estrogen receptor beta ,Fatty acid binding protein adipocyte,Fatty acid binding protein epidermal ,Fatty acid binding protein muscle ,Fatty acid-binding protein-liver, Formyl peptide receptor 1 ,Glucocorticoid receptor ,Glucose-6-phosphate 1-dehydrogenase ,Glutamate NMDA receptor; GRIN1/GRIN2B ,G-protein coupled bile acid receptor 1,G-protein coupled receptor 55 ,Heme oxygenase 1 (by homology) ,Histone deacetylase 8 .HMG-CoA reductase ,IgG receptor FcRn large subunit p51 ,Low molecular weight phosphotyrosine protein phosphatase , LSD1/CoREST complex ,LXR-alpha ,Mineralocorticoid receptor ,Muscarinic acetylcholine receptor M2 ,Niemann-Pick C1-like protein 1 ,Norepinephrine transporter ,NT-3 growth factor ,Nuclear factor NF-kappa-B p105 subunit ,Nuclear receptor ROR-alpha ,Nuclear receptor subfamily 1 group I member 3 (by homology) ,Peroxisome proliferator-activated receptor alpha ,Peroxisome proliferator-activated receptor delta ,Phospholipase A2 group 1B ,Potassium-transporting ATPase alpha chain 2 ,Pregnane X receptor ,Prelamin-A/, Progesterone receptor ,Protein-tyrosine phosphatase 1B ,Protein-tyrosine phosphatase 1C, Receptor-type tyrosine-protein phosphatase F (LAR) ,Serotonin 2c (5-HT2c) receptor ,Serotonin transporter , Sigma opioid receptor ,Squalene monooxygenase ,Sterol regulatory element-binding protein 2 ,T-cell protein-tyrosine phosphatase ,Testis-specific androgen-binding protein ,Transcription intermediary factor 1-alpha ,Transient receptor potential cation channel subfamily M member 8 ,Transient receptor potential cation channel subfamily V member 3 ,Voltage-gated N-type calcium channel alpha-1B subunit. |
| LINALOOL | 5-hydroxytryptamine receptor 3A ,Adrenergic receptor alpha-2, Alpha-1a adrenergic receptor ,Androgen Receptor ,Bloom syndrome protein ,Cannabinoid CB2 receptor ,Carbonic anhydrase I ,Carbonic anhydrase II ,Carbonic anhydrase III ,Carbonic anhydrase IV ,Cathepsin D ,Cyclooxygenase-2 ,Delta opioid ,Dipeptidyl peptidase IX ,Dipeptidyl peptidase VIII ,DNA-(apurinic or apyrimidinic site) lyase ,Dopamine D2 receptor (by homology) ,Dopamine transporter (by homology) ,Estradiol 17-beta-dehydrogenase ,Glucocorticoid receptor ,Glycine transporter ,G-protein coupled bile acid receptor 1 ,G-protein coupled receptor 35 ,Heme oxygenase 1 (by homology) ,Histamine H3 receptor ,Histamine H4 receptor ,IgG receptor FcRn large subunit p51 ,Indoleamine 2,3-dioxygenase ,Kappa Opioid receptor ,Kruppel-like factor 5. |
| MYRCENE | Peroxisome proliferator-activated receptor alpha ,Cannabinoid receptor 2, MAP kinase ERK2 ,Tyrosyl-DNA phosphodiesterase 1 ,DNA-(apurinic or apyrimidinic site) lyase ,Nuclear factor NF-kappa-B p105 subunit ,Dual specificity protein kinase CLK4 ,Endoplasmic reticulum-associated amyloid betapeptide-binding protein ,Cathepsin D ,Glycine transporter 2 ,Histone deacetylase 3 ,Monoamine oxidase A ,Carbonic anhydrase III ,Dipeptidyl peptidase IX ,Dipeptidyl peptidase VIII ,G-protein coupled receptor 35 ,Tyrosine-protein kinase TEC ,Pregnane X receptor .Epidermal growth factor receptor erbB1 ,Geranylgeranyl pyrophosphate synthetase. |
| VERBENONE | 11-beta-hydroxysteroid dehydrogenase 1 ,11-beta-hydroxysteroid dehydrogenase 2 ,Acyl coenzyme A:cholesterol acyltransferase P23141 CES1 , Adenosine A3 receptor ,Alcohol dehydrogenase alpha chain, Alcohol dehydrogenase gamma chain ,Aldo-keto reductase family 1 member B10 ,Anandamide amidohydrolase ,Androgen Receptor ,Arachidonate 5-lipoxygenase ,Beta-secretase 1 ,Butyrylcholinesterase ,Cannabinoid CB2 receptor ,Carboxylesterase 2 ,Casein kinase II alpha/beta ,Cathepsin D ,C-C chemokine receptor type 5 ,Corticosteroid binding globulin ,Cyclooxygenase-1 ,Cytochrome P450 17A1 ,Cytochrome P450 19A1 P11511 ,DNA topoisomerase II alpha ,DNA-(apurinic or apyrimidinic site) lyase ,Dual specificity phosphatase Cdc25A ,Dual specificity protein kinase CLK4 ,Dual specificty protein kinase CLK1 ,Estradiol 17-beta-dehydrogenase 3 ,Estrogen receptor alpha ,Estrogen receptor beta ,Fatty acid-binding protein, liver (by homology) ,Glucocorticoid receptor ,G-protein coupled receptor 55 ,Kruppel-like factor 5 , LSD1/CoREST complex ,MAP kinase ERK1 |
