# Supplementary material for: In Vitro Evaluation and Network Pharmacology Analysis of the Antimicrobial Activity of Pistacia lentiscus
Source: Int J Dent. 2026 Jan 20;2026:6981413. doi: 10.1155/ijod/6981413 (PMC12817137; doi:10.1155/ijod/6981413)
Supplement: Supplementary file 2 — Supporting Information 2 Figure A: Protein–protein interaction network retrieved from STRING database for the compound‐specific targets‐alpha‐terpineol, linalool, myrcene, verbenone. Figure B: Functional enrichment analysis: alpha‐terpineol A. Biological process B. Molecular process C. Cellular process D. KEGG pathway analysis. Figure C: Functional enrichment analysis: linalool A. Biological process B. Molecular process C. Cellular process D. KEGG pathway analysis. Figure D: Functional enrichment analysis: verbenone A. Biological process B. Molecular process C. Cellular process D. KEGG pathway analysis. Figure E: Functional enrichment analysis: myrcene ‐ KEGG pathway analysis. Figure F: cytoscape‐verbenone, myrcene, linalool. Figure G: CFU counts on agar plates-Pseudomonas aeuroginosa. Figure H: CFU counts on agar plates‐Escherichia coli. Figure I: CFU counts on agar plates‐Staphylococcus aureus. Figure J: CFU counts on agar plates‐Enterococcus hirae. Figure K: CFU counts on SDA plates‐Aspergillus niger. Figure L: CFU counts on SDA plates‐Candida albicans. [file IJOD-2026-6981413-s002.docx]

SUPPLEMENTARY FIGURES


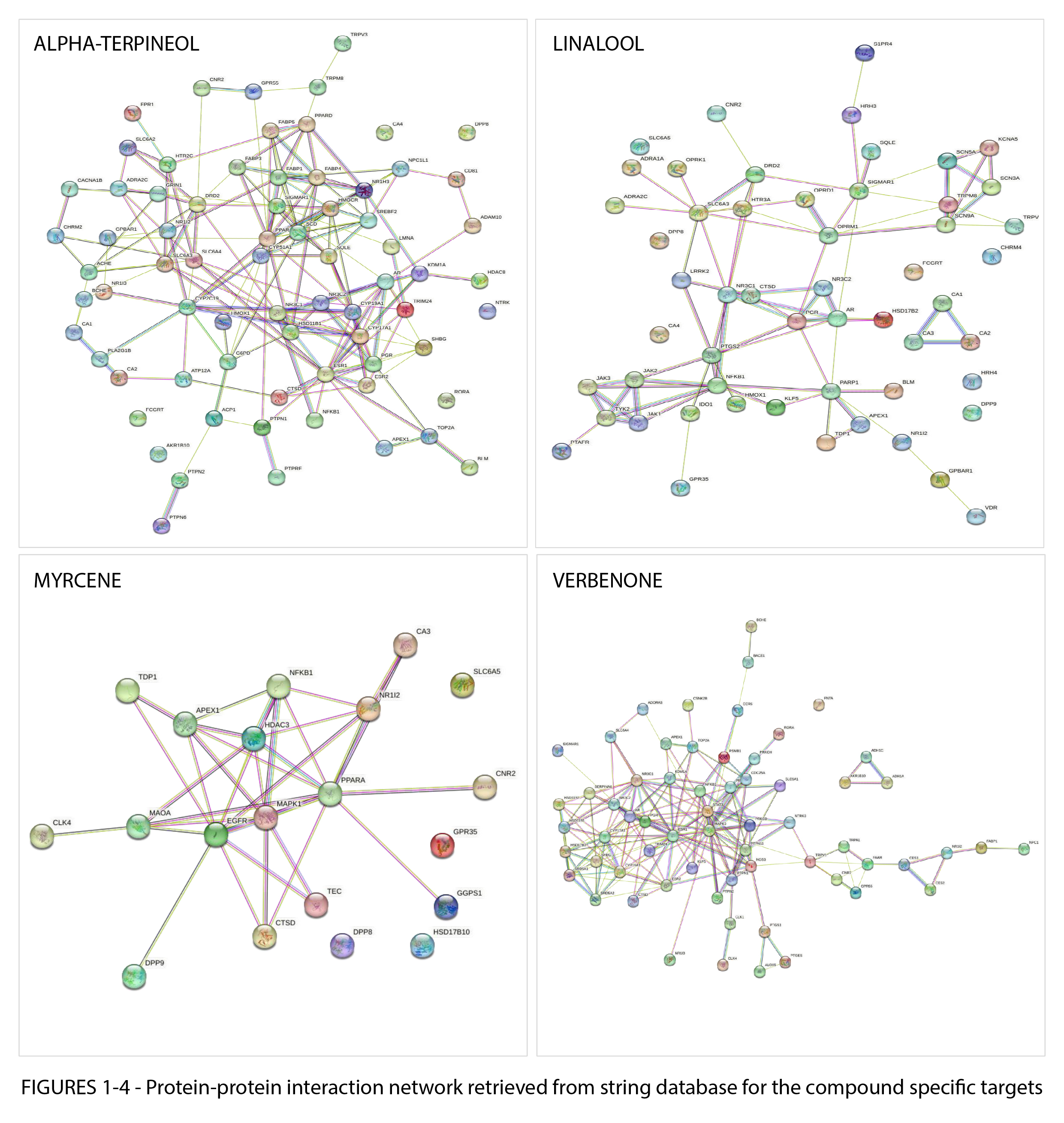


FIGURE A: Protein-protein interaction network retrieved from string database for the compound specific targets-ALPHA TERPINEOL,LINALOOL,MYRCENE,VERBENONE


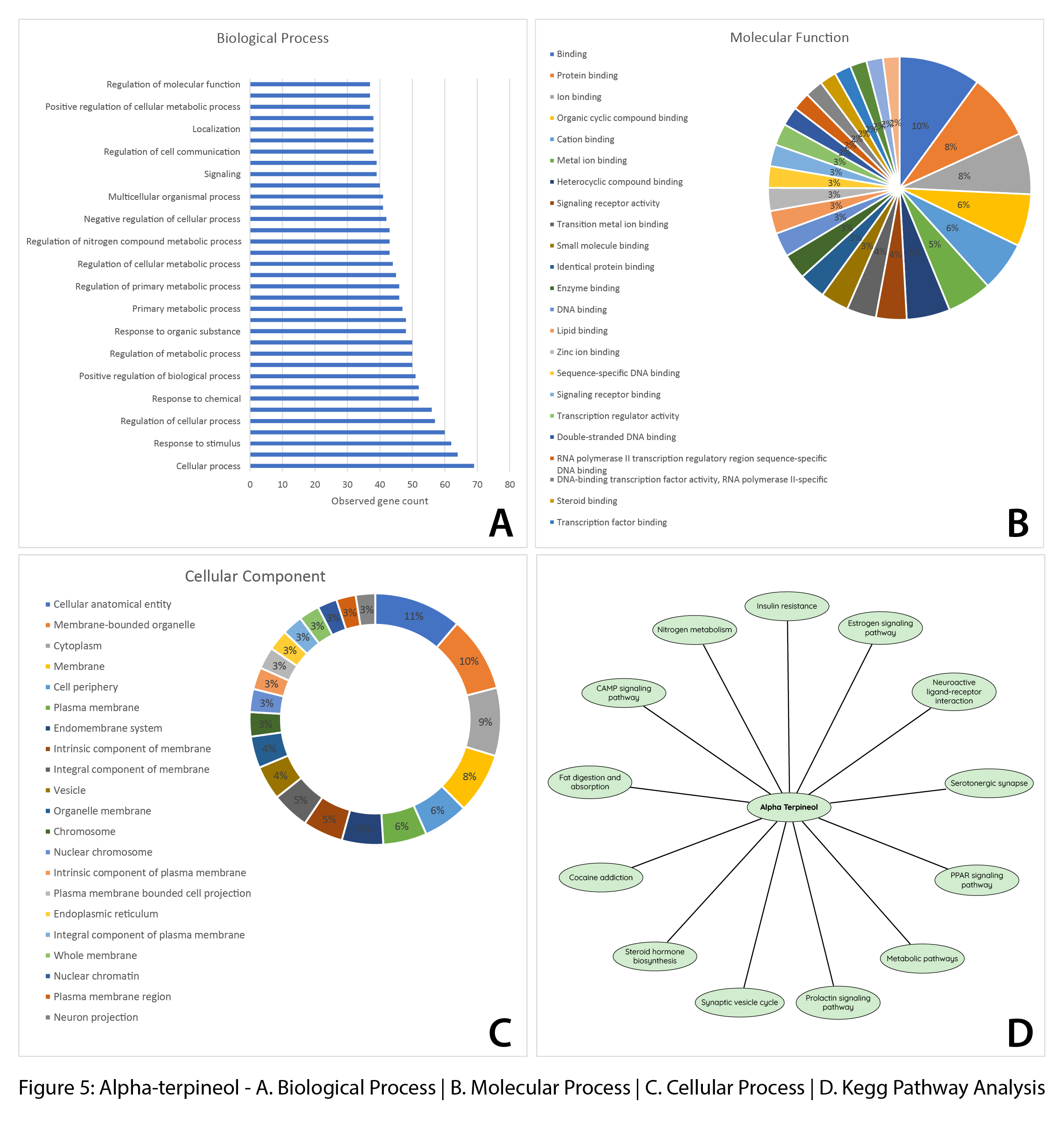


FIGURE B: FUNCTIONAL ENRICHMENT ANALYSIS: ALPHA-TERPINEOL -A. BIOLOGICAL PROCESS B. MOLECULAR PROCESS C. CELLULAR PROCESS D. KEGG PATHWAY ANALYSIS


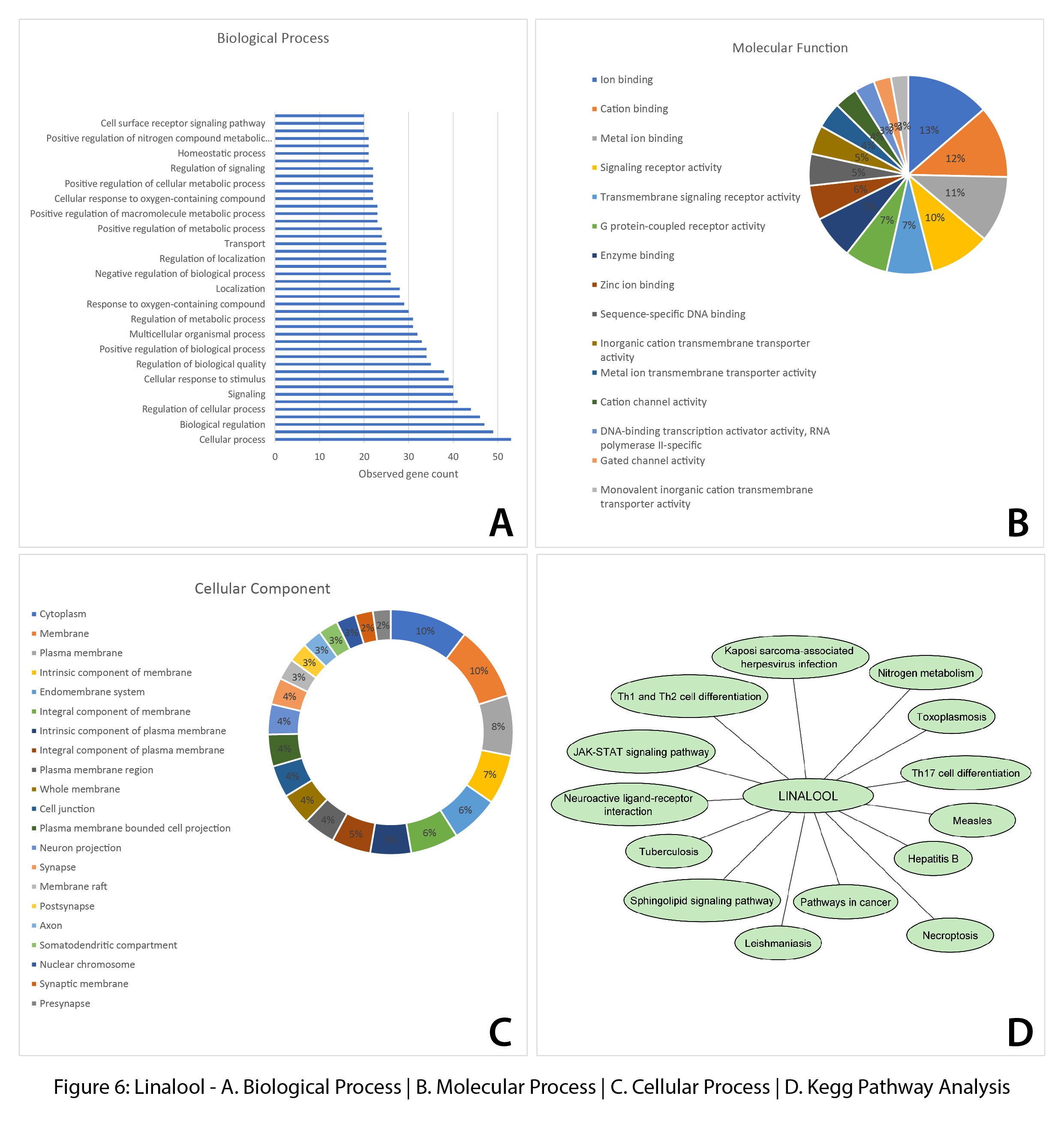


FIGURE C: FUNCTIONAL ENRICHMENT ANALYSIS: LINALOOL -A. BIOLOGICAL PROCESS B. MOLECULAR PROCESS C. CELLULAR PROCESS D. KEGG PATHWAY ANALYSIS


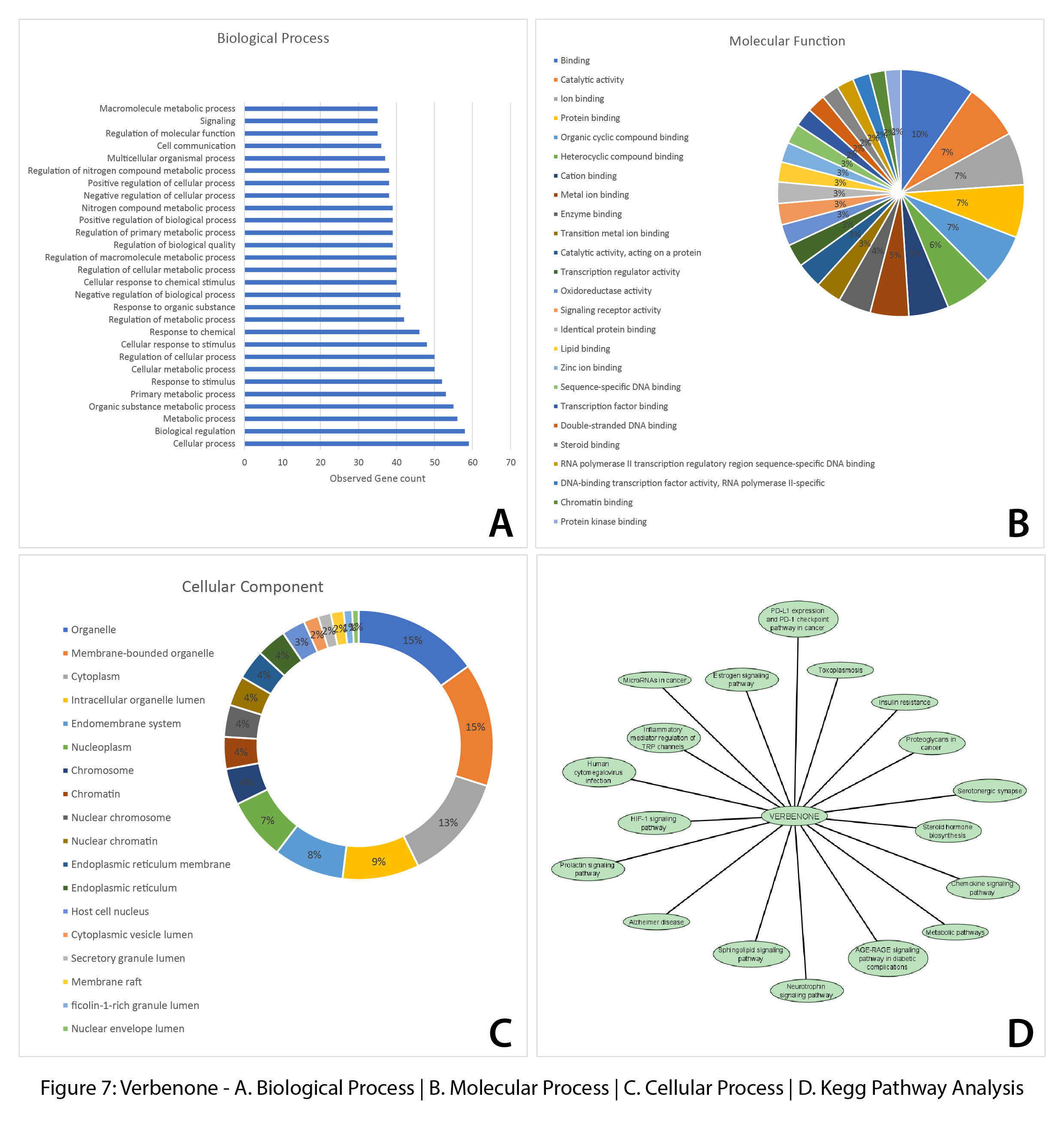


FIGURE D: FUNCTIONAL ENRICHMENT ANALYSIS: VERBENONE -A. BIOLOGICAL PROCESS B. MOLECULAR PROCESS C. CELLULAR PROCESS D. KEGG PATHWAY ANALYSIS


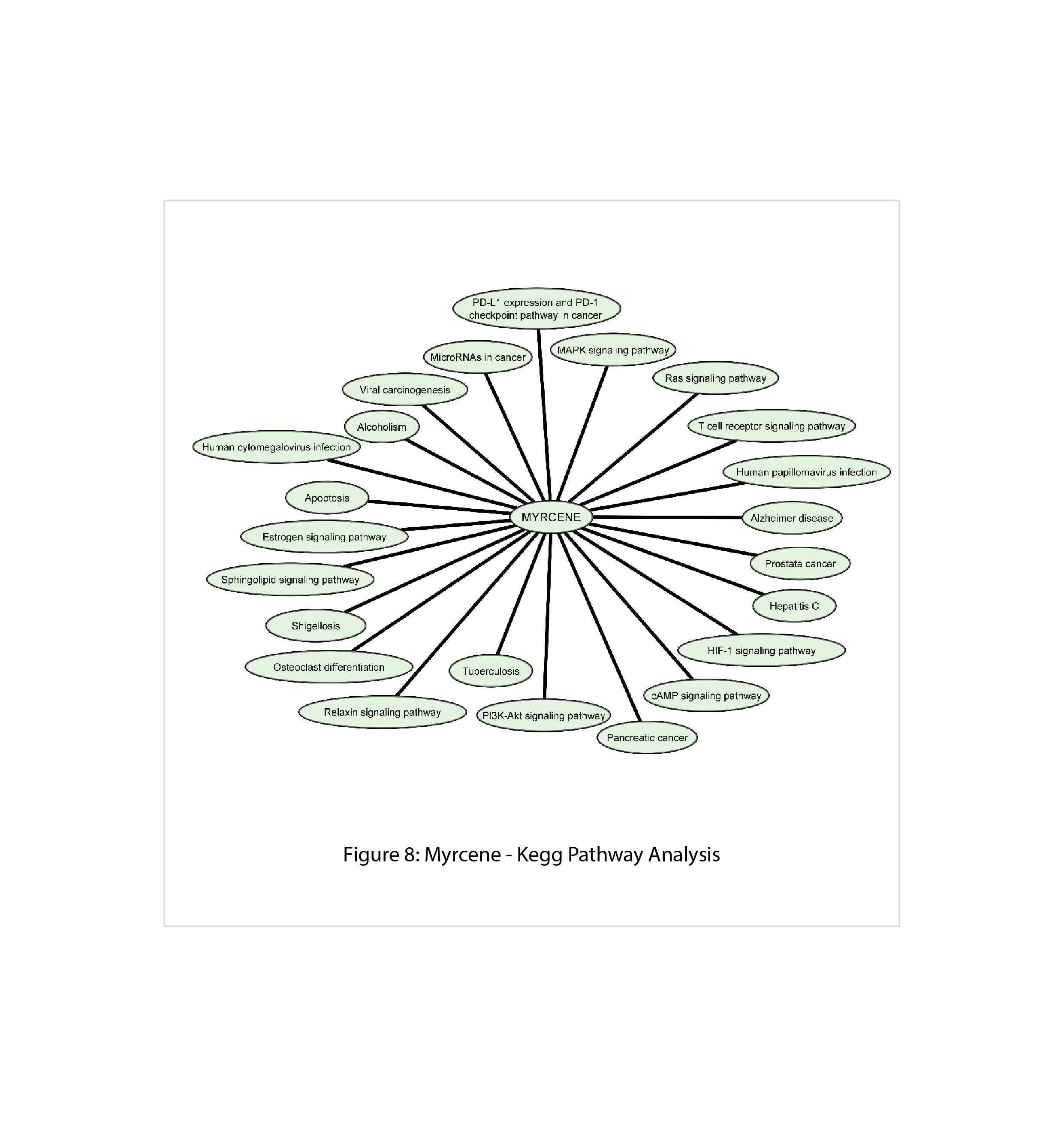


FIGURE E: FUNCTIONAL ENRICHMENT ANALYSIS: MYRCENE - KEGG PATHWAY ANALYSIS


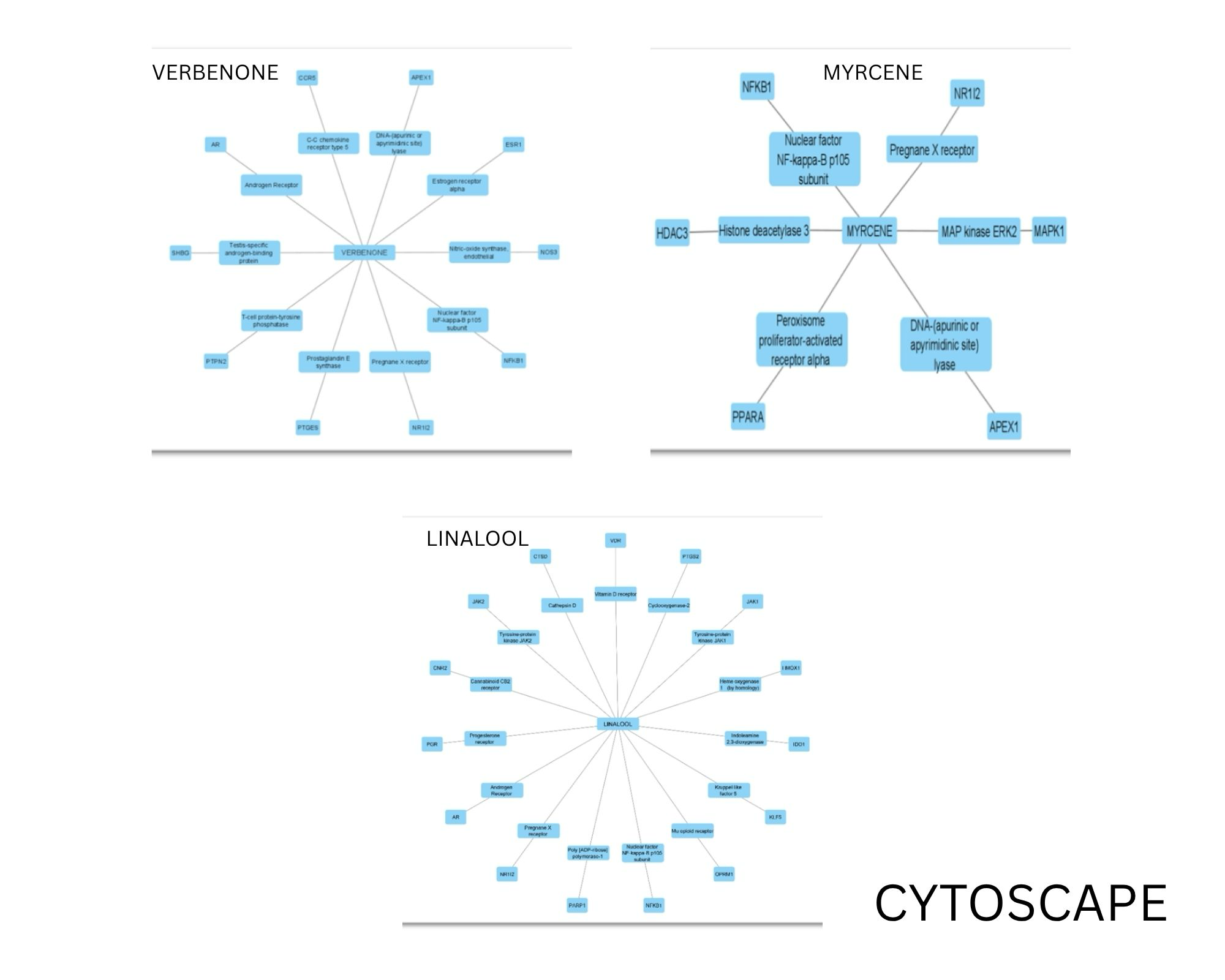


FIGURE F: CYTOSCAPE-VERBENONE,MYRCENE,LINALOOL


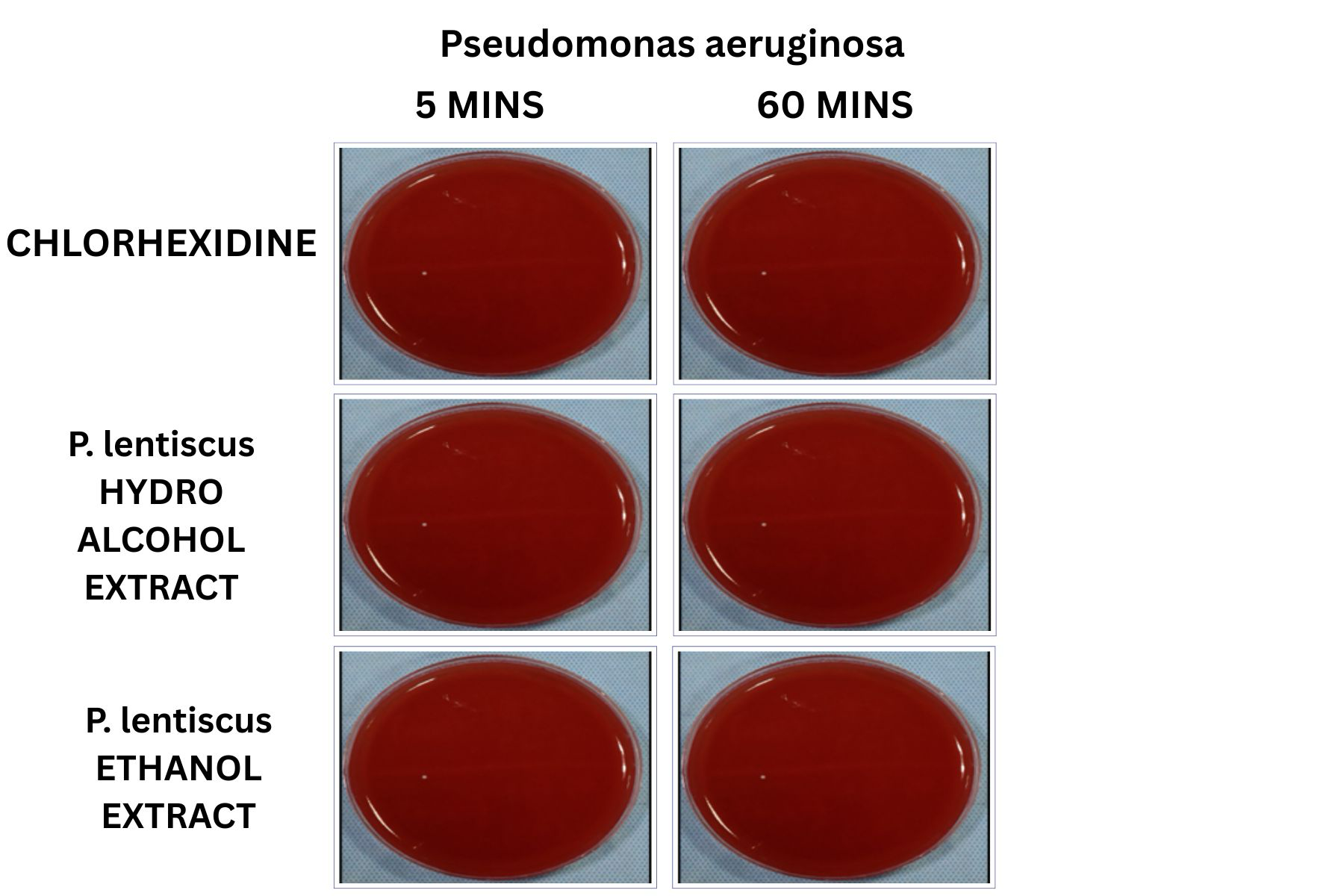


FIGURE G:CFU COUNTS ON AGAR PLATES-*Pseudomonas aeuroginosa*


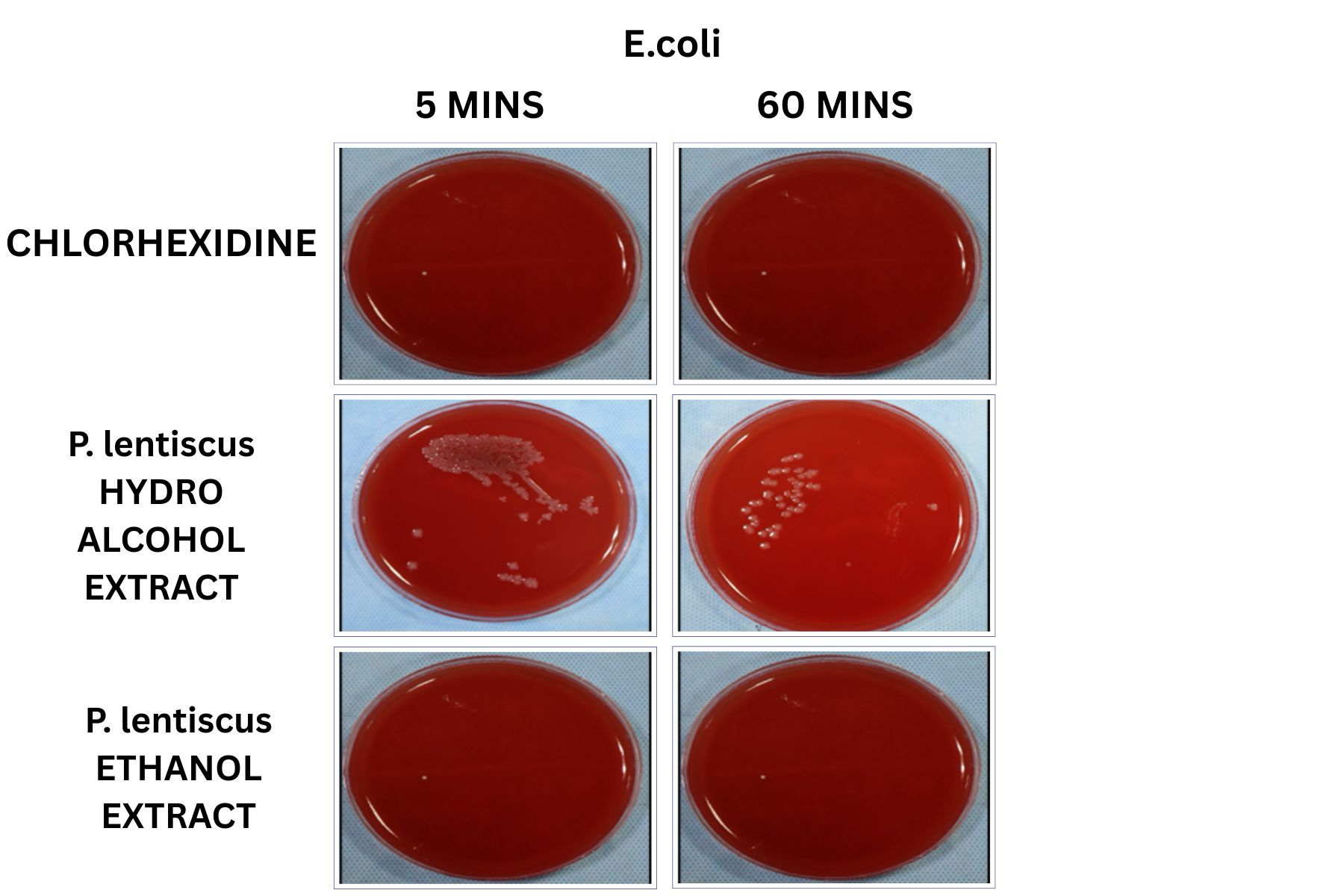
 FIGURE H: CFU COUNTS ON AGAR PLATES-*Escherichia coli*


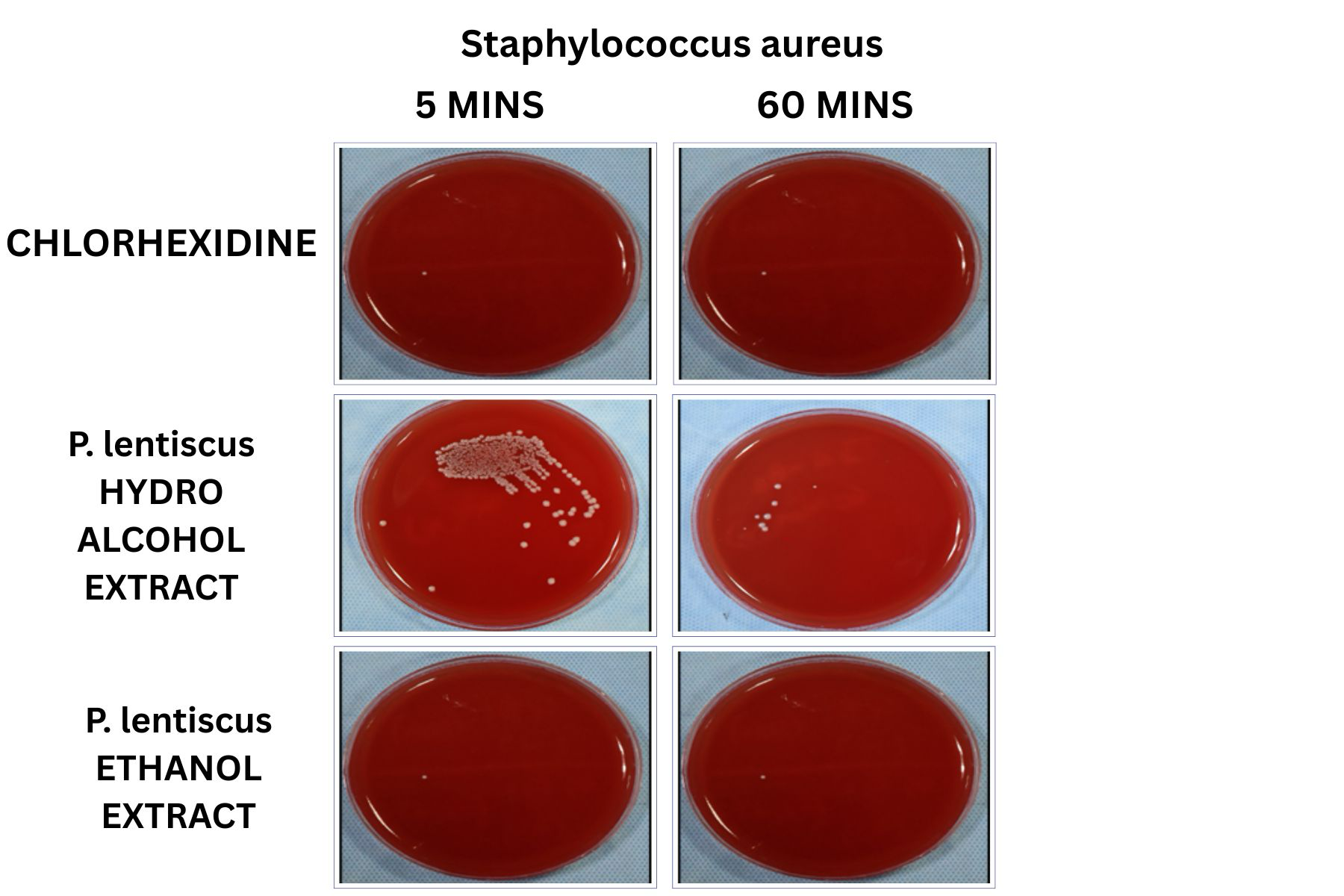


FIGURE I: CFU COUNTS ON AGAR PLATES-*Staphylococcus aureus*


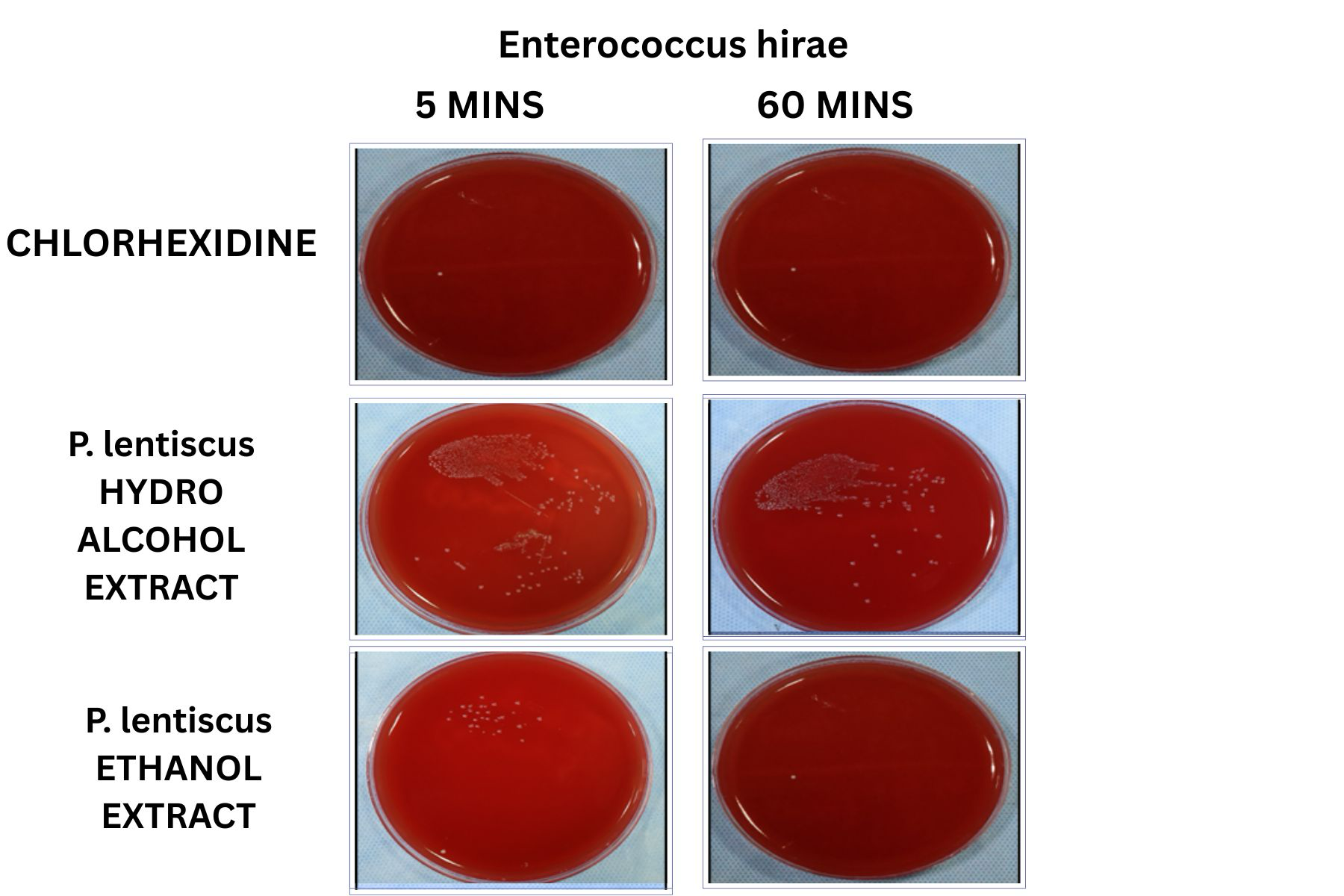


FIGURE J: CFU COUNTS ON AGAR PLATES-*Enterococcus hirae*


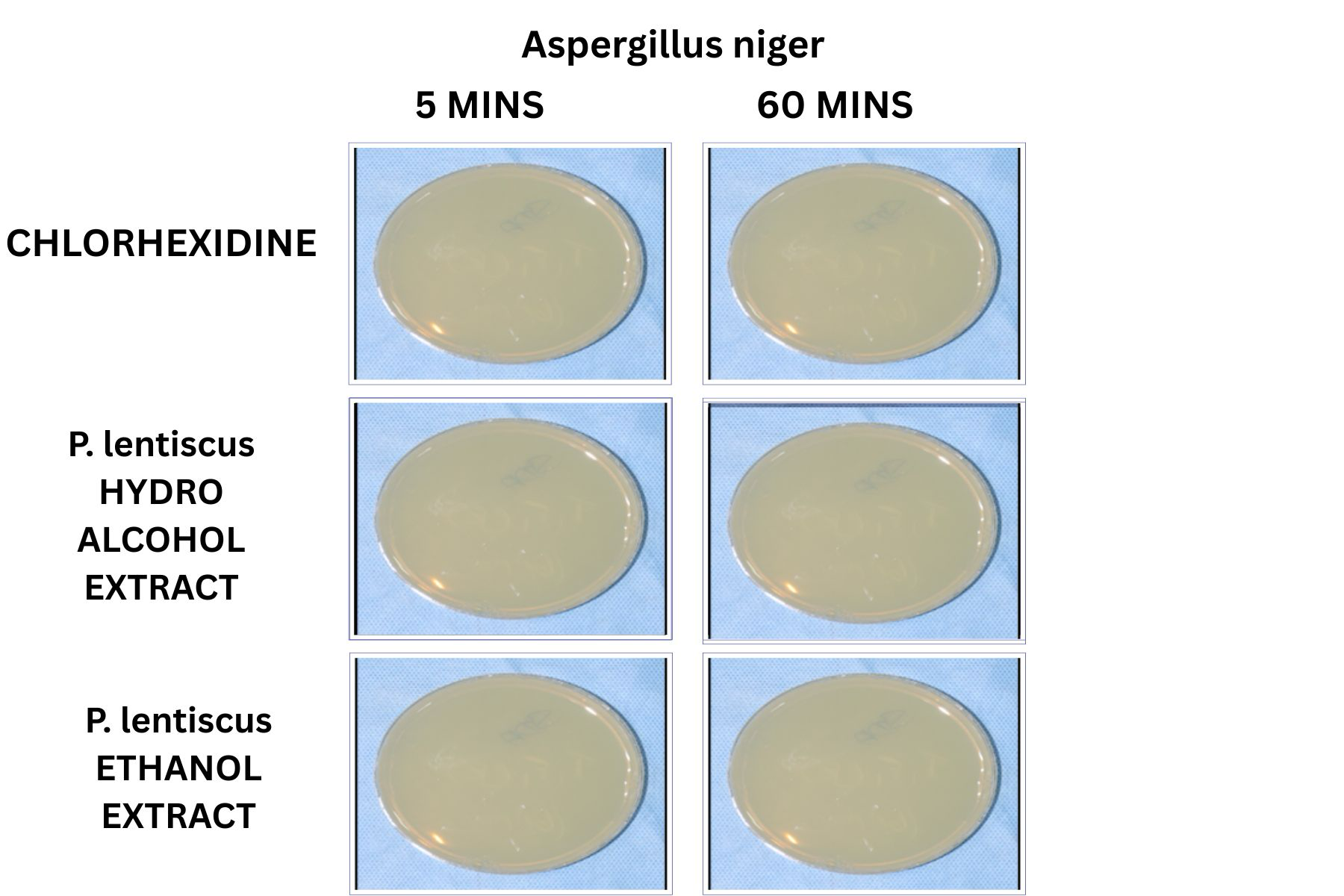
 FIGURE K:CFU COUNTS ON SDA PLATES-*Aspergillus niger*


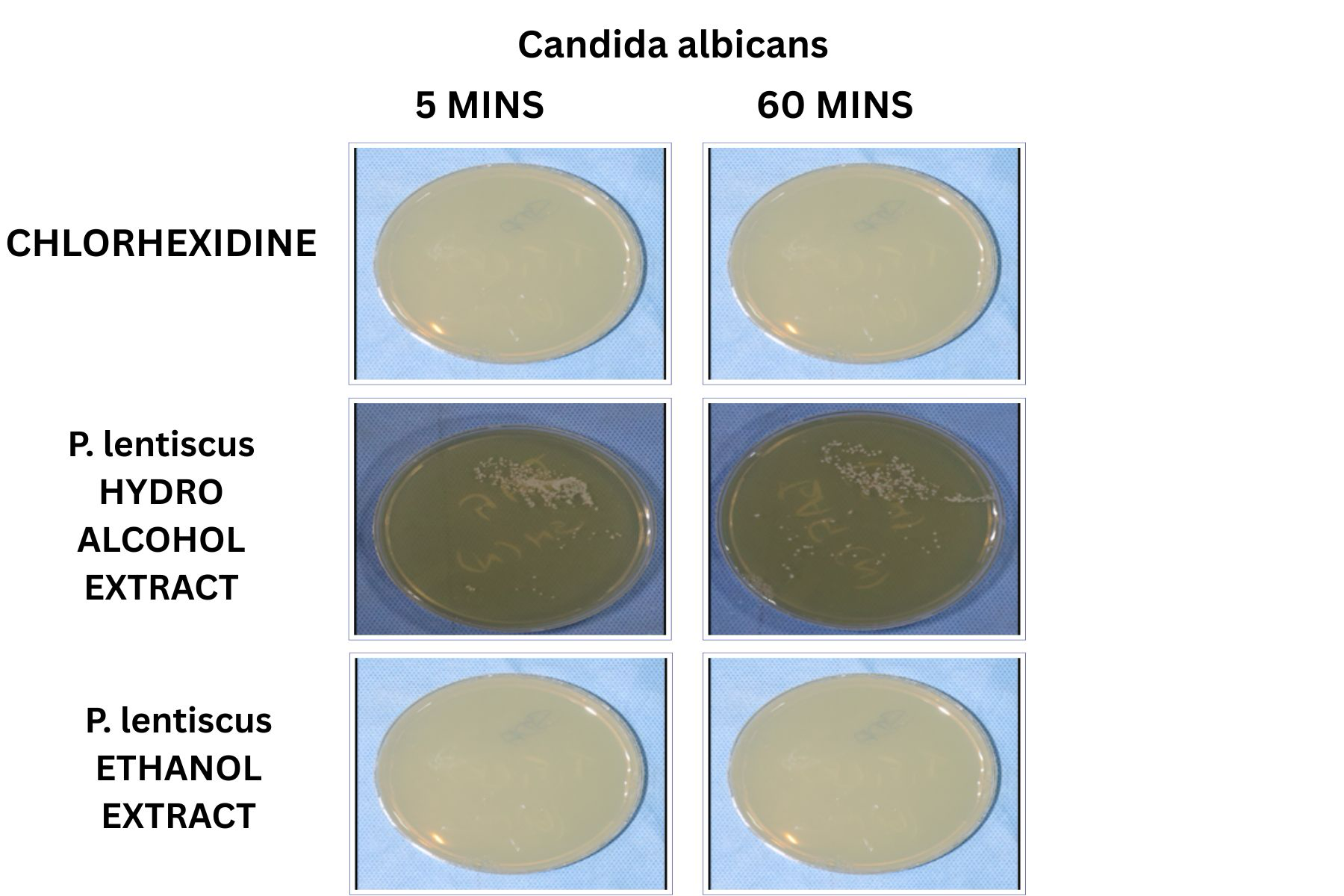


FIGURE L:CFU COUNTS ON SDA PLATES-*Candida albicans*
